# Train Like You Compete? Physical and Physiological Responses on Semi-Professional Soccer Players

**DOI:** 10.3390/ijerph17030756

**Published:** 2020-01-24

**Authors:** Alfonso Castillo-Rodríguez, Francisco Javier Cano-Cáceres, António Figueiredo, José Carlos Fernández-García

**Affiliations:** 1 Department of Physical Education and Sports, University of Granada, 18010 Granada, Spain; javiercano@correo.ugr.es; 2 University of Coimbra, Centro de Investigaçao do Desporto e da Atividade Física, Faculdade de Ciências do Desporto e Educaçao Física, 3030 Coimbra, Portugal; afigueiredo@fcdef.uc.pt; 3 Universidad de Málaga, Andalucía-Tech, IBIMA, 29010 Malaga, Spain; jcfg@uma.es

**Keywords:** soccer, training, competition, match, physical responses, physiological responses

## Abstract

**Background:** Decision-making in soccer has repercussions and depends on the environment of training or competition. The demands on the players can reveal if the decision-making is similar or different from that required during competition. **Objectives:** The aim of this study was to assess the physical and physiological responses of players in training matches (TM) and official competition matches (CM) according to the playing position (external defenders, internal defenders, midfielders, and forwards/extremes). **Methods:** Twenty semi-professional male soccer players and 10 CM (n = 40) and 10 TM (n = 40) were studied using global positioning system technology, and paired and one-way ANOVA tests were carried out to compare physical (distances and number of sprints) and physiological (heart rates) responses with the factors a) match environments (TM and CM) and b) the playing position, respectively. **Results:** The results revealed that during CM, players covered higher total distance, partial distances, and sprints at different speeds (0–21 km/h) and produced higher physiological responses. Midfielders covered the greatest total distance in both TM (7227.6 m) and CM (11,225.9 m), in comparison to the other playing positions. However, forwards and extremes spent more time (56.8% of the CM [d = 0.78]) at 76% to 84% of their maximal heart rates. **Conclusions:** First, the physical and physiological responses in TM were significantly lower than in CM. Second, these responses were different according to the playing position, so this study was able to verify the exact amount of variation between the load produced in TM and CM. These results will help the coach and technical staff to design training tasks to complement the responses found in TM.

## 1. Introduction

To achieve good sports performance, it is necessary that athletes and coaches take into account aspects related to biomechanics, psychology, physical condition, and technical-tactical aspects, among others [1]. Decision-making is a variable that determines how, when, how much and why an athlete performs an action or behaviour. For this reason, this focus of interest is currently a topic of discussion and research that has impacts on both training and competition. A fundamental aspect is that the athlete can make correct decisions and thus perform optimal physical, technical, and tactical actions [2]. In this case, the impulsivity of the athlete can predetermine the decision-making. It is known that defensive players can be characterized as less impulsive and more concentrated and premeditated than players with an offensive role, so it is estimated that the physical and physiological responses can be differentiated according to the role being played [3].

Currently, soccer is one of the most popular sports in the world, practised by 300 million persons with more than 1.7 million teams throughout the world [4,5]. This popularity has resulted in a considerable rise in the number of research studies with the goal of improving the understanding of demands on players to optimize training time. 

To this end, in order to understand the physical and physiological demands required of the soccer player during training matches (TM) and competition matches (CM), global positioning system (GPS) devices were used to measure speed, with high reliability and validity [6]. It has been seen that in TM, the soccer player spends more time at lower levels of speed, while, in CM, more time is spent at higher speeds [7]. Thus, the maximum speed achieved during TM and CM was different. Additionally, different physical responses such as total distance (TD) covered and physiological responses such as heart rate (HR) depend on the playing position [8,9]. Midfielders are players whose position of play is midway between the attacking forwards and the defenders, and they cover higher TD during CM [10].

Soccer matches require different periods of activity that vary in intensity and duration and alternate between periods of incomplete recuperation and scarce activity [11]. In this sense, the decrease in fatigue and increase in sport performance are the objective of training, and the process of recuperation plays an important role. The effect of these training sessions will vary depending on the physical and physiological responses [12].

On the other hand, the level of the teams seems to be determined by the TD covered by the players. In the Premier League, the lowest ranked teams cover more TD at high-speed than do the highest ranked teams [13]. These authors demonstrated that the technical-tactical aspect prevails over physical-physiological responses in terms of sports success. Similarly, repeated sprints sequences depend on the playing position, with a range between 1000–1400 activity changes [14]. Physiologically, midfielders report higher mean heart rate (meanHR) and internal defenders demand lower internal load, in general [1,15]. These studies concluded that players occupying central positions in the field are subjected to higher internal and external loads. The coaches and physical trainers play an important role in attempting to reproduce the physical and physiological demands on players during competition, with the end goal of attaining sport success [16].

In this way, understanding the physical and physiological demands on soccer players will enable the optimization of individual and team performance, thereby determining the level of play [17]. This information is relevant for coaches, physical trainers, and technical staff, in general, as well as for the players themselves, helping them prepare for future matches, could determine their individual needs [18], and more importantly, understand technical staff decisions in the preparation of the training sessions. Therefore, the aim of this study is to assess the physical and physiological responses in TM and CM according to the playing position on semi-professional soccer players.

## 2. Methods

### 2.1. Subjects

Twenty semi-professional soccer players (age 24.7 ± 3.9 years, height 181.4 ± 6.0 cm, and weight 77.9 ± 7.0 kg) were the subjects of the present study, which was conducted in the middle of soccer season (weeks 18–32 of 42). The players had an average age that started playing football at a competitive level of 8.4 ± 2.2 years, being 5 and 12, the lower and upper starting ages. All of the players were members of the same team (Third Division in Spain). In total, 10 matches were recorded in each context (40 TM and 40 CM). They were classified into 4 groups based on their playing position: external defenders (ED), internal defenders (ID), midfielders (MI), and forward/extremes (FO), pursuant to the classification established in the studies of Cardenas-Fernandez et al. [19] and Lago–Penas et al. [20] All subjects were informed of the objectives and procedures and completed the corresponding voluntary consent form pursuant to the guidelines established by the Declaration of Helsinki (2016) [21]. The University of Granada Ethics Committee approved the implementation of this study (471/CEIH/2018).

### 2.2. Instruments

#### 2.2.1. Physical Demands

GPS technology (SPI-PRO devices, GPSports, Canberra, Australia) along with Team AMS 1.2 software was used in this study. These devices operate at 5 Hz and its validation was performed by Petersen et al. [22] Five categories were established for speed movements, in sprints (S) and in longer distances (D), adopting the criteria established in other studies, but adding a last interval that values sprints over 21 km/h [23]: walking from 0.1 to 7.0 km/h (distances [DW] and number of sprints [SW] covered by walking), running at low-speed from 7.1 to 13.0 km/h (distances [DL] and number of sprints [SL] covered by low-speed), running at medium-speed from 13.1 to 18.0 km/h (distances [DM] and number of sprints [SM] covered by medium-speed), running at high-speed from 18.1 to 21.0 km/h (distances [DH] and number of sprints [SH] covered by high-speed), and sprinting at >21.0 km/h (distances [DS] and number of sprints [SS] covered by sprinting). Moreover, the TD, mean speed, and maximum speed were also collected as physical responses. 

#### 2.2.2. Physiological Demands

HR was measured using Polar S610i devices (Polar Electro Oy, Helsinky, Finland). Four categories were established based on theoretical maximum HR (maxHR) [23]: HR1 (HR ≤ 75%), HR2 (HR between 76 and 84%), HR3 (HR between 85 and 89%), and HR4 (HR > 90%). Minimum HR (minHR), meanHR, and maxHR values for each half of the match were obtained by graphic analysis (Polar Precision Performance 5.0 and Team AMS 1.2).

### 2.3. Procedures

This study used a transversal observational method in order to examine the internal and external loads of the soccer players during CM and TM through GPS technology. Four players were monitored for each TM and for each CM. Ten matches per playing position were recorded. All matches took place in outdoor installations with artificial grass fields and with 11 players on each team. Tactically, the team used a 1-5-3-2 formation, with a variation of 1-4-4-2. The time period recorded was 2 × 45 min. Data for time movement was included only for the players who participated in the entire match. The opposing teams were all at a similar level. Four players were monitored for each TM and for each CM. Ten matches per playing position were recorded. The procedure carried out was as follows. First, a warm-up was developed, exactly the same in duration (volume) and intensity in both contexts. Then, they had a few minutes rest between the warm-up and the match so that the head coach could indicate the basic and specific notions or rules of play. During TM, the coach did not stop the match to explain faults in the play, just as in CM. The rest time carried out between the 2 halves was the same (15 min). 

### 2.4. Statistical Analyses

The statistical package SPSS for Windows version 22 (IBM SPSS Statistic, Chicago, USA) and Microsoft Office Excel (Microsoft Corp., Redmond, Washington, DC, IL, USA) were used. The Shapiro-Wilks test was used to verify normality of variables. Although some variables did not follow a normal distribution, the sample size in each group was adequate (n ≥ 20) to apply the central boundary theorem, which provides normally distributed sample means [24]. Therefore, parametric analyses were performed in this study. A test of repeated measures (paired test) was used to assess the difference between the CM and TM, and subsequently, one-way analysis of variance (ANOVA) was run on the internal and external load variables in relation to playing position. To evaluate the difference between distinct comparisons between groups, a Bonferroni post hoc test was performed. The threshold values for the Cohen effect sizes detected in a t-test (*d*) are 0.20 for small effects, 0.50 for moderate effects, and 0.80 for large effects. For ANOVA tests, those values (*η^2^*) were 0.10 for small effects, 0.25 for moderate effects, and 0.40 for large effects [25]. The level of significance was established at *p* < 0.05.

## 3. Results

First, the differences between the TM and CM were analysed. Table 1 shows the values of the physiological response of the players in both CM and TM. There are multiple differences between both environments, except for the variable HR2 (*p* > 0.05). In CM, the physiological responses were greater, except in HR1 (*p* = 0.001; *d* = 0.55).

Furthermore, the physical responses of the players in both CM and TM are shown in Table 2. Multiple differences were found between both environments, in particular, there were significant differences in the TD, DW, DL, DM, DH, DS, SW and SL variables. The remaining variables did not show significant differences (*p* > 0.05). The physical responses in the CM were higher, except for the maximum speed variable, and the SS variable had similar values in both environments.

On the other hand, taking into account the general differences between TM and CM, the responses of the players in the matches were analysed according to the playing position and the match environments. Table 3 shows the values of the physiological responses of the soccer players in both CM and TM according to the playing positions. There were multiple differences between different playing positions, except for the minHR, meanHR, and maxHR variables (*p* < 0.05). In the CM, the physiological responses were greater, except in the HR1 and HR2 variables, where it was lower in the CM. For all variables, external defenders could be detected as those players with a higher HR, both in the TM and in CM.

Table 4 shows the values on the physical responses of the players both in the CM and TM according to the playing positions. It can be observed that the physical responses in the CM are greater, except on the external defender players in the DW, DS, SW, and SS variables; on the internal defender players in DL, DS, SW, and SL; on the midfielder players in DH, DS, SM, SH, and SS; and on the forwards in DW, DS, SW, and SS. The position with a higher TD was the midfielder, in TM (7227.6 m) and CM (11225.9 m). Finally, those with a maximum speed were the forward players, in TM (27.12 km/h) and CM (29.02 km/h).

## 4. Discussion

The aim of this study was to assess the physical and physiological responses of semi-professional soccer players in TM and CM depending on their playing positions. In this sense, the scientific literature confirms that players demand different internal and external load between training days and official-competition matches [7,8,9]. These demands may also vary according to the position occupied by the soccer players on the pitch based on the system designed by the coaches, and whether or not the team has possession of the ball [26].

On the other hand, during high-intensity actions, e.g., DH, DS, SH, and SS, among others, we can observe that they follow the same trend, with higher values in CM with respect to TM. Considering these results, the responses during TM by the same players are not replicated during CM, possibly due to the environment of the training, player cohesion, and satisfaction with the role on the pitch [27]. This is evident from the degree of arousal activation in players during CM [28], and the inclusion of situation of small-sided games in the trainings at present [29]. These tasks do not allow reaching high-intensity speeds or sprinting (>21 km/h) because of the evident reduced dimensions of the space [23].

The monitoring of distances and speeds has been carried out through studies in several sports, such as soccer [23], orienteering [30], athletics [31,32], cricket [33], rugby [34,35], and tennis [36,37], accepting the reliability of the GPS, and its practical application in most team sports, since errors are relatively scarce and predictable [38]. In addition, the devices used in this study show high reliability in order to measure TD and the distances developed at both low and medium speed [33].

Regarding TD during TM and CM by semi-professional players according to their playing positions, significant differences are observed for all positions (*p* < 0.05). Hartwig et al. [9], similarly to our study, found differences in the playing positions. The MI position covers the highest TD, both in TM and CM; the FO position achieves the highest maximum speed, both in TM and in CM; defensive players covered the lowest TD in both environments [14]. In addition, it is estimated that differences are found for all playing positions in all sub-categories of sprint in both environments (except in SS). This leads to the conclusion that the playing position has an important role in the influence of the sprint development [39]. This information reveals the specific demands during CM in semi-professional soccer players to prescribe specific trainings reproducing repeated sprint sequences, race distances and speed peaks [39], adapted to the individual demands of the players based on tactical training [40].

Nevertheless, the partial distances covered show a remarkable difference produced by the specific functions of players with different roles. Internal defenders showed lower values of distance at high-speed, while forwards and midfielders presented the highest values of TD, and, physiologically, the midfielder playing position had the lowest meanHR, measured in beats per minute (bpm). However, for the HR4 variable measured in percentage of time, the midfielder players have a value much higher than the rest of positions, becoming the position with the highest physiological demand. On the other hand, the wide differences between internal defenders and midfielders observed in this study could be related to tactical roles and training adaptations. The midfielder players have a liaison role in the team and need to complete quickly medium distance movements compared to the defending players, who practice more planned movements and demand a high concentration [41], with more sensible or prudent decisions than players with a bigger offensive role. Di Salvo et al. [12] indicated that the midfielder has functions to take advantage of the scoring opportunities while they must return to defend themselves when their team loses ball possession. Therefore, coaches should know that players do not train like they compete, and players need more attention during a TM to optimize physical and physiological performance during a CM [42]. For example, the encouragement of technical staff in different parts of the field, the fast inclusion of balls when the game is stopped or modifying the existence of the offside could cause an increase in these demands in a real game.

Certain limitations of this study should be considered. First, the sample size could have been larger in order to generalize the results to a large population such as semi-professional soccer players. In Spain, there are 300 semi-professional teams and a total of 6000 football players. To generalize the results, it would be necessary to propose a future project where teams from different areas are involved. On the other hand, the limitation in the number of GPS devices meant that the number of matches analyzed was greater, which implies that, in this study, it is very important to consider the size of the effect and even the possible coefficient of variation of the dependent variables. In this sense, knowing that the effect size is high, the conclusions have been established from this indicator. Finally, it could compare the responses produced both in their own field and in the opposite field in order to offer more information to coaches and athletes. In this sense, the teams that faced each other were taken into account; there were no notable differences in terms of the general classification, which would have been a contaminating variable.

## 5. Conclusions

The main finding of this study is that responses during CM are significantly higher than those obtained during TM. Physical (covered distances and sprints) and physiological responses (heart rate) developed by soccer players were different. Therefore, soccer players do not train as they compete. In addition, midfielder and forward players have the highest physical and physiological demands, and less so for internal defenders, which confirms different features influenced by the role assumed in the playing field.

## Figures and Tables

**Table 1:** Paired tests of physiological responses of soccer players in competition matches (CM) and training matches (TM).

	CM (N = 40)	TM (N = 40)	*p*	*d*
minHR (bpm)	116.1 ± 14.3	102.1 ± 18.5	0.025	0.53
meanHR (bpm)	164.0 ± 7.7	152.3 ± 13.0	0.004	0.48
maxHR (bpm)	186.2 ± 8.0	178.3 ± 9.8	0.021	0.46
HR1 (% time)	10.2 ± 8.0	33.9 ± 27.4	0.001	0.55
HR2 (% time)	32.0 ± 17.5	35.7 ± 20.4	0.592	0.10
HR3 (% time)	26.7 ± 9.1	16.1 ± 10.6	0.006	0.48
HR4 (% time)	31.1 ± 24.3	14.3 ± 24.1	0.070	0.33

*p*: significant value; *d*: effect size; CM: competition match; TM: training match; bpm: beats per minute; minHR: minimum heart rate; meanHR: mean heart rate; maxHR: maximum heart rate; HR1: heart rate covered until 75% (inclusive) of the theoretical maxHR; HR2: heart rate covered from 76% until 84% of the theoretical maxHR; HR3: heart rate covered from 85% until 89% of the theoretical maxHR; HR4: heart rate covered greater than 90% of the theoretical maxHR.

**Table 2:** Paired tests of physical responses of soccer players in CM and TM.

	CM (N = 40)	TM (N = 40)	*p*	*d*
meanS (km/h)	6.8 ± 0.6	6.5 ± 1.0	0.331	0.36
maxS (km/h)	28.2 ± 2.3	28.6 ± 3.6	0.698	0.41
TD (m)	10,022.7 ± 1180.0	6213.2 ± 933.1	0.000	0.80
DW (m)	4010.5 ± 509.9	2578.5 ± 220.9	0.000	0.61
DL (m)	3467.9 ± 713.7	2120.9 ± 653.8	0.000	0.51
DM (m)	1755.0 ± 542.3	1004.6 ± 407.2	0.002	0.42
DH (m)	457.3 ± 146.6	293.0 ± 127.3	0.011	0.34
DS (m)	332.2 ± 134.3	216.3 ± 107.1	0.030	0.15
SW (number)	426.8 ± 46.4	343.7 ± 75.7	0.001	0.57
SL (number)	616.8 ± 73.1	487.2 ± 136.5	0.002	0.53
SM (number)	292.1 ± 65.7	239.1 ± 92.5	0.072	0.32
SH (number)	98.4 ± 19.5	91.3 ± 38.4	0.496	0.12
SS (number)	29.0 ± 6.6	28.9 ± 13.7	0.993	0.01

*p*: significant value; *d*: effect size; CM: competition match; TM: training match; meanS: mean speed; maxS: maximum speed; TD: Total distance; DW and SW: distance covered and number of sprints run between 0 and 6.9 km/h; DL and SL: distance covered and number of sprints run between 7.0 and 12.9 km/h; DM and SM: distance covered and number of sprints run between 13.0 and 17.9 km/h; DH and SH: distance covered and number of sprints run between 18.0 and 20.9 km/h; DS and SS: distance covered and number of sprints run from 21.0 km/h.

**Table 3:** One-way ANOVA of physiological responses of soccer players in TM and CM according to the playing position.

		ED (N = 10)	ID (N = 10)	MI (N = 10)	FO (N = 10)	*F_(1,39)_*	*p*	*d*
minHR (bpm)	TM	104.6 ± 19.3	106.4 ± 26.9	108.7 ± 29.2	95.4 ± 23.8	0.442	0.725	0.15
	CM	112.0 ± 10.6	119.1 ± 4.6	116.3 ± 33.3	108.2 ± 13.7	0.294	0.829	0.12
meanHR (bpm)	TM	150.7 ± 28.3	147.6 ± 32.3	145.8 ± 38.3	139.6 ± 29.8	0.173	0.914	0.09
	CM	163.3 ± 6.1	161.8 ± 3.0	151.2 ± 39.7	152.7 ± 1.9	0.477	0.702	0.16
maxHR (bpm)	TM	177.1 ± 32.0	171.1 ± 36.9	162.2 ± 42.7	165.0 ± 35.2	0.274	0.844	0.12
	CM	190.0 ± 7.5	187.9 ± 8.0	166.7 ± 41.5	181.4 ± 11.1	1.150	0.359	0.24
HR1 (% time)	TM	20.4 ± 22.6	14.3 ± 15.8	7.1 ± 7.8	33.5 ± 26.0	2.408	0.089	0.33
	CM	9.6 ± 6.9 ^FO^	5.8 ± 4.2 ^FO^	3.5 ± 3.6 ^FO^	19.7 ± 5.1 ^ED,ID,MI^	8.200	0.002	0.55
HR2 (% time)	TM	28.9 ± 5.7	31.6 ± 16.3	22.4 ± 21.8 ^FO^	49.8 ± 18.0^MI^	4.151	0.015	0.42
	CM	29.0 ± 4.5 ^MI,FO^	22.8 ± 6.5 ^FO^	14.5 ± 9.7 ^ED,FO^	56.8 ± 8.7 ^ED,ID,MI^	28.906	0.000	0.78
HR3 (% time)	TM	29.1 ± 10.4 ^FO^	26.4 ± 7.9	19.2 ± 8.4	15.0 ± 11.0 ^ED^	3.681	0.024	0.40
	CM	33.6 ± 5.1 ^FO^	28.9 ± 7.3	21.8 ± 9.7	20.7 ± 9.0 ^ED^	3.271	0.050	0.38
HR4 (% time)	TM	21.7 ± 13.0 ^MI^	27.7 ± 21.2	51.4 ± 30.5 ^ED,FO^	1.8 ± 3.9 ^ED,MI^	8.649	0.000	0.56
	CM	27.8 ± 10.0 ^MI,FO^	42.5 ± 12.9 ^FO^	60.2 ± 22.7 ^ED,FO^	2.8 ± 4.8 ^ED,ID,MI^	15.139	0.000	0.66

*p*: significant value; *d*: effect size; CM: competition match; TM: training match; bpm: beats per minute; minHR: minimum heart rate; meanHR: mean heart rate; maxHR: maximum heart rate; HR1: heart rate covered until 75% (inclusive) of the theoretical maxHR; HR2: heart rate covered from 76% until 84% of the theoretical maxHR; HR3: heart rate covered from 85% until 89% of the theoretical maxHR; HR4: heart rate covered greater than 90% of the theoretical maxHR; ED: external defender; ID: internal defender; MI: midfielder; FO: forward. Bonferroni post hoc (*p* < 0.05) with playing position as the exponent.

**Table 4:** One-way ANOVA of physical responses of soccer players in TM and CM according to playing position.

		ED (N = 10)	ID (N = 10)	MI (N = 10)	FO (N = 10)	*F_(1,39)_*	*p*	*d*
meanS (km/h)	TM	5.6 ± 1.2	5.7 ± 1.2	6.4 ± 1.7	6.2 ± 1.4	0.696	0.563	0.19
	CM	6.1 ± 0.4	6.2 ± 0.3	6.5 ± 1.7	6.7 ± 0.6	0.378	0.770	0.14
maxS (km/h)	TM	26.9 ± 5.3	25.8 ± 5.6	23.7 ± 5.5	27.1 ± 3.9	0.782	0.514	0.19
	CM	28.5 ± 2.3	28.3 ± 1.8	24.1 ± 6.2	29.0 ± 1.4	2.138	0.136	0.32
TD (m)	TM	5275.6 ± 786.5	5790.3 ± 393.1	7227.6 ± 53.0	6559.2 ± 951.3	3.501	0.129	0.40
	CM	9369.0 ± 1042.8	9697.7 ± 431.4	11,225.9 ± 673.3	10,104.8 ± 1491.5	2.710	0.080	0.36
DW (%)	TM	45.2 ± 7.3 ^MI^	45.2 ± 4.6 ^MI^	31.2 ± 4.1 ^ED,ID^	37.6 ± 7.8	8.401	0.000	0.55
	CM	41.3 ± 4.0	45.9 ± 5.5 ^MI^	32.0 ± 4.2 ^ID^	36.1 ± 7.5	5.153	0.012	0.46
DL (%)	TM	31.2 ± 4.3 ^MI^	35.9 ± 2.9	38.9 ± 4.2 ^ED^	34.9 ± 5.4	4.391	0.012	0.43
	CM	33.6 ± 2.3	35.0 ± 1.9	39.6 ± 5.0	35.2 ± 6.1	1.721	0.205	0.29
DM (%)	TM	15.1 ± 3.0 ^MI^	13.7 ± 2.9 ^MI^	21.5 ± 3.8 ^ED,ID^	18.1 ± 3.6	7.811	0.001	0.53
	CM	16.6 ± 2.3	13.9 ± 4.1 ^MI^	21.8 ± 4.9 ^ID^	19.4 ± 3.5	3.749	0.034	0.40
DH (%)	TM	4.6 ± 0.8 ^ID^	3.0 ± 0.7 ^ED,MI,FO^	5.2 ± 1.8 ^ID^	5.6 ± 1.0 ^ID^	7.692	0.001	0.54
	CM	4.9 ± 0.4	3.1 ± 0.8^FO^	4.7 ± 1.9	5.6 ± 0.5 ^ID^	4.748	0.016	0.45
DS (%)	TM	3.9 ± 1.3	2.3 ± 0.9	3.1 ± 2.5	3.9 ± 1.6	1.702	0.190	0.29
	CM	3.6 ± 1.0	2.2 ± 0.5	2.0 ± 1.1 ^FO^	3.8 ± 1.5 ^MI^	3.425	0.045	0.39
SW (%)	TM	71.9 ± 6.0 ^MI^	69.1 ± 3.7 ^MI^	56.1 ± 4.2 ^ED,ID^	64.4 ± 8.2	10.348	0.000	0.59
	CM	68.6 ± 2.8 ^MI^	69.0 ± 3.7 ^MI^	56.8 ± 3.5 ^ED,ID^	63.1 ± 8.6	5.135	0.012	0.46
SL (%)	TM	19.5 ± 4.1 ^MI^	23.5 ± 2.7	29.6 ± 3.0 ^ED^	24.2 ± 5.8	7.800	0.001	0.54
	CM	21.8 ± 2.0 ^MI^	23.4 ± 1.8	29.7 ± 3.5 ^ED^	24.7 ± 6.5	3.320	0.049	0.38
SM (%)	TM	6.2 ± 1.7 ^MI^	5.9 ± 1.5 ^MI^	11.1 ± 2.4 ^ED,ID^	8.4 ± 2.4	10.590	0.000	0.59
	CM	7.0 ± 1.1 ^MI^	6.1 ± 2.0 ^MI^	11.0 ± 2.8 ^ED,ID^	9.1 ± 2.4	4.968	0.014	0.45
SH (%)	TM	1.5 ± 0.3	1.0 ± 0.2 ^MI,FO^	2.2 ± 1.0 ^ID^	2.0 ± 0.6 ^ID^	6.742	0.002	0.51
	CM	1.6 ± 0.1	1.0 ± 0.3 ^FO^	1.9 ± 0.9	2.0 ± 0.4 ^ID^	3.673	0.036	0.40
SS (%)	TM	1.0 ± 0.4	0.6 ± 0.3	1.1 ± 0.9	1.1 ± 0.5	1.572	0.219	0.28
	CM	1.0 ± 0.3	0.6 ± 0.2	0.7 ± 0.4	1.1 ± 0.4	2.724	0.081	0.35

*p*: significant value; *d*: effect size; CM: competition match; TM: training match; meanS: mean speed; maxS: maximum speed; TD: Total distance; DW and SW: distance covered and time of sprints run between 0 and 6.9 km/h; DL and SL: distance covered and time of sprints run between 7.0 and 12.9 km/h; DM and SM: distance covered and time of sprints run between 13.0 km/h and 17.9 km/h; DH and SH: distance covered and time of sprints run between 18.0 and 20.9 km/h; DS and SS: distance covered and time of sprints run from 21.0 km/h; ED: external defender; ID: internal defender; MI: midfielder; FO: forward. Bonferroni post hoc (*p* < 0.05) with playing position as the exponent.
